# Potential use of V-channel Ge(220) monochromators in X-ray metrology and imaging

**DOI:** 10.1107/S0021889813006122

**Published:** 2013-06-07

**Authors:** D. Korytár, P. Vagovič, K. Végsö, P. Šiffalovič, E. Dobročka, W. Jark, V. Áč, Z. Zápražný, C. Ferrari, A. Cecilia, E. Hamann, P. Mikulík, T. Baumbach, M. Fiederle, M. Jergel

**Affiliations:** a Institute of Electrical Engineering, SAS, Dúbravská cesta 9, 841 04 Bratislava, Slovakia; b ANKA Light Source, Karlsruhe Institute of Technology, Karlsruhe, Germany; c Institute of Physics, SAS, Dúbravská cesta 9, 845 11 Bratislava, Slovakia; d Sincrotrone Trieste ScpA, Basovizza (TS), Italy; e Alexander Dubček University of Trenčín, Študentská 2, SK-911 50 Trenčín, Slovakia; f CNR IMEM Institute, Viale Usberti 37/A, Parma, 43124, Italy; g CEITEC, Masaryk University, Kotlářská 2, CZ-61137 Brno, Czech Republic; h FMF, Freiburg, Germany

**Keywords:** metrology, Montel optics, Bragg magnifier, channel-cut crystal

## Abstract

While channel-cut crystals, in which the diffracting surfaces in an asymmetric cut are kept parallel, can provide beam collimation and spectral beam shaping, they can in addition provide beam compression or expansion if the cut is V-shaped. The compression/expansion ratio depends in this case on the total asymmetry factor. If the Ge(220) diffraction planes and a total asymmetry factor in excess of 10 are used, the rocking curves of two diffractors will have a sufficient overlap only if the second diffractor is tuned slightly with respect to the first one. This study compares and analyses several ways of overcoming this mismatch, which is due to refraction, when the Cu *K*α_1_ beam is compressed 21-fold in a V21 monochromator. A more than sixfold intensity increase was obtained if the matching was improved either by a compositional variation or by a thermal deformation. This provided an intensity gain compared with the use of a simple slit in a symmetrical channel-cut monochromator. The first attempt to overcome the mismatch by introducing different types of X-ray prisms for the required beam deflection is described as well. The performance of the V-shaped monochromators is demonstrated in two applications. A narrow collimated monochromatic beam obtained in the beam compressing mode was used for high-resolution grazing-incidence small-angle X-ray scattering measurements of a silicon sample with corrupted surface. In addition, a two-dimensional Bragg magnifier, based on two crossed V15 channel monochromators in beam expansion mode and tuned by means of unequal asymmetries, was successfully applied to high-resolution imaging of test structures in combination with a Medipix detector.

## Abstract 2

Several ways of tuning a higher asymmetry factor (>10) in V-channel X-ray monochromators, for metrological and imaging applications, were analysed. A more than sixfold intensity increase for compositionally and thermally tuned cases was achieved.

## 1. Introduction

With progress in materials science and technology, X-ray sources and beam conditioning optics that can provide high brightness and high resolution in reciprocal and real space are all the more important for the metrology of advanced micro- and nanostructures. Standard laboratory high-resolution X-ray diffractometry with a Göbel mirror and Bartels monochromator can now be, in many cases, successfully replaced by an X-ray microsource, Montel optics and crystal optics adjusted to required parameters (degree of beam monochromaticity, collimation and beamsize). A detailed analysis of the performance of various optics in common experimental setups was presented by Fewster (2000 ▶). A special group of Bragg case channel-cut crystals with nonparallel channel walls (V-shaped monochromators) offering beam footprint control (one-dimensional beam expansion or compression) was discussed by Pietsch *et al.* (2004 ▶) and dealt with in detail by Korytár *et al.* (2008 ▶). Similar to all two-bounce channel-cut monochromators, these are in-line monochromators characterized by parallel input and output beams. Because of the unequal refraction angle shifts at the channel walls, the overlap of the two rocking curves inside the channel decreases if the asymmetry angles increase. Consequently, the intensity transmitted through the monochromator decreases significantly for total asymmetry factors greater than 10.

The idea of using two crossed asymmetric diffractions for imaging purposes has been known for a long time (Boettinger *et al.*, 1979 ▶; Stampanoni *et al.*, 2002 ▶). Crossed V-channel monochromators were successfully demonstrated recently as Bragg magnifying imaging optics in combination with a FReLoN camera (Vagovič *et al.*, 2011 ▶).

This paper presents our theoretical and experimental study on how higher asymmetry V-channel monochromators can be tuned. We analyzed various approaches and compared parameters of such devices. Two practical applications are also demonstrated: (1) a beam compressing V-channel monochromator in a grazing-incidence small-angle X-ray scattering (GISAXS) system for use in metrology and (2) an imaging application.

## 2. Theoretical background

**Figure 1:**
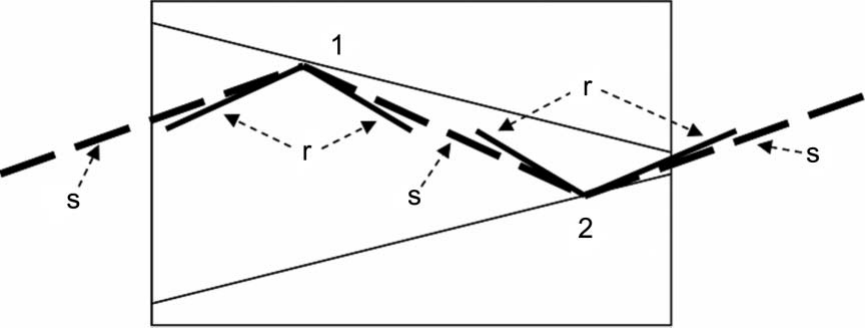
Sketch of a V21 CCM with an asymmetry angle α = 15°. Long dashed s lines represent the incident and diffracted X-ray beams in a kinematic approximation with a zero refraction correction; shorter r beams represent refraction-corrected directions (angular deviations exaggerated).

In symmetric and asymmetric channel-cut monochromators with parallel channel walls the refraction angle shifts are compensated for and rocking curves fully overlapped. By contrast, in V-channel monochromators, refraction shifts are added at the nonparallel walls and the overlap of rocking curves is less (Korytár *et al.*, 2010 ▶). Fig. 1 ▶ shows kinematic angularly unshifted (s lines) and refraction-corrected (r lines) X-ray beams inside a V-channel; the total angular deviation that needs to be corrected is given roughly as the sum of the two refraction corrections inside the channel:
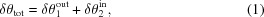
where
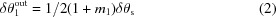
and 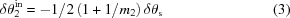
are the output refraction corrections from the first and the input from the second diffractor, respectively.

Refraction correction (1) does not need to be fully corrected because it is related to the centre of the rocking curves. It is sufficient to correct the refraction correction decreased by a value that is slightly less than the sum of halves of the rocking curves δω_tot_, namely
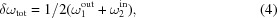
where
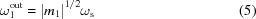
and 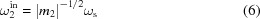
are the output divergence from the first and the input acceptance of the second diffractor, respectively. Here 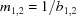 is the magnification factor, where
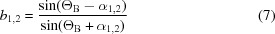
are the asymmetry factors, and Θ_B_ and α_1,2_ are the Bragg angle and the asymmetry angles of the two diffractors, respectively. The total de/magnification from both diffractors is 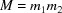. For the other symbols see, for example, Hrdý (2001 ▶). 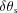 and 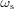 are the corresponding refraction correction and the rocking curve width values for the symmetrical case, respectively.

Equations (1)–(7) [Disp-formula fd3] [Disp-formula fd4] [Disp-formula fd5] [Disp-formula fd6] [Disp-formula fd7] work reliably for many applications except extremely asymmetric cases with the angles of incidence or exit close to the critical angle and except when the Bragg angle is close to 90°. More complicated expressions for the refraction corrections for the angles of incidence close to the critical angle and the corresponding angular widths of rocking curves are presented by Ferrari *et al.* (2011 ▶). A general approach based on the dynamical theory covering these cases also was presented by Shvyd’ko (2004 ▶) and, recently, the spectral dispersion was dealt with in detail by Huang *et al.* (2012 ▶). The other tools that are used for the design of monochromators and complete optical schemes are rocking curve calculations (http://sergey.gmca.aps.anl.gov/), du Mond diagrams (Shvyd’ko, 2004 ▶) and the beam tracing programs *XOP*, *RAY* (Schäfers, 2008 ▶) and *SKL* (Mikulík & Kuběna, 2005 ▶).

**Figure 2:**
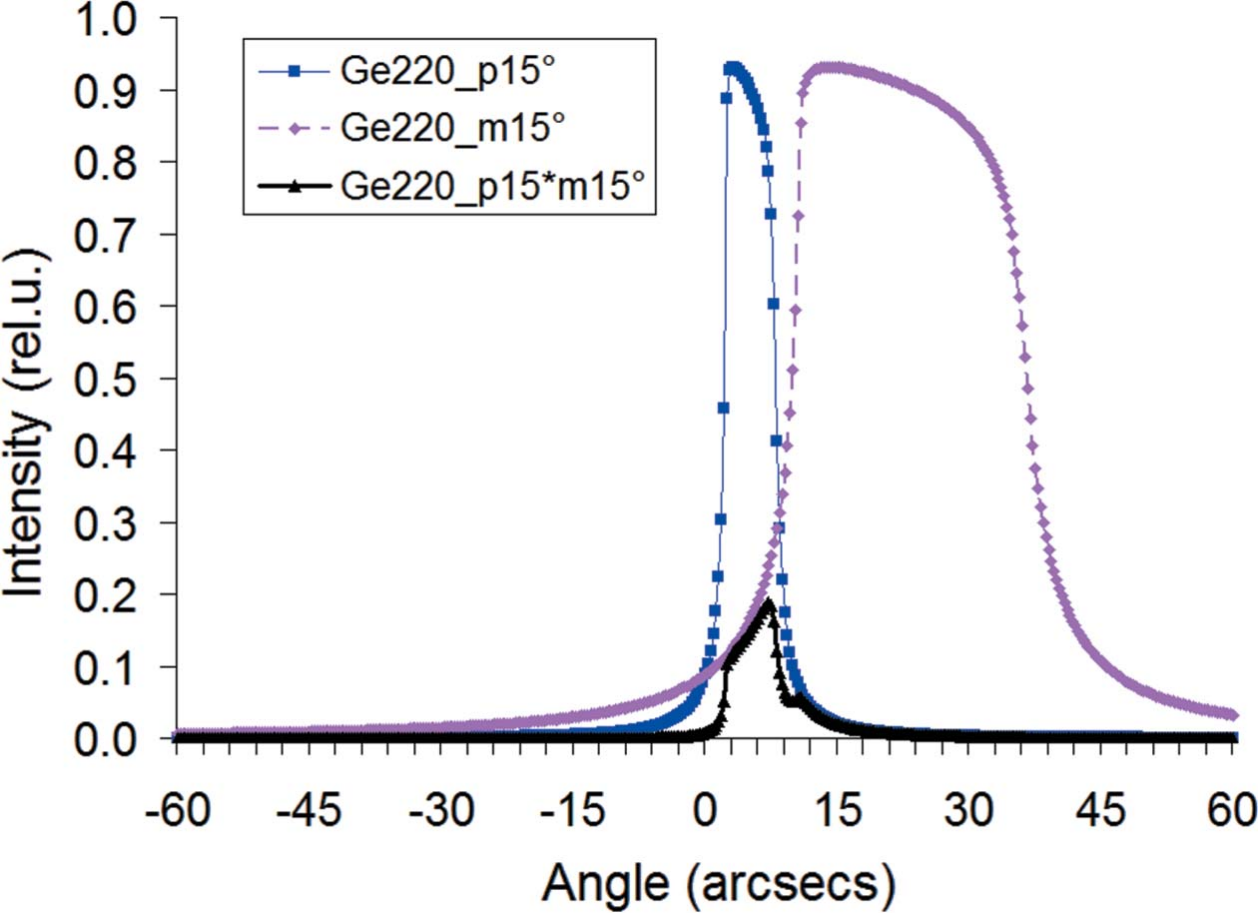
Rocking curve diffracted at position 1 (narrower one) inside the channel of the V21 monochromator, the acceptance rocking curve at position 2 and their product (both for beam expander and compressor).

The equations for coupling the successive diffractors were presented by Korytár *et al.* (2008 ▶). Fig. 1 ▶ shows a sketch of a Ge(220) V-channel monochromator for Cu *K*α radiation, namely a V21 monochromator with an asymmetry angle α = 15°. Contrary to the kinematic approximation (s-beams), the dynamical theory gives a nonzero refraction correction according to equations (1)–(3) [Disp-formula fd3], which is different for the incident and outgoing beams (and also different angular widths); the differences increase with asymmetry angles (r-beams in Fig. 1 ▶). Fig. 2 ▶ shows rocking curves inside the channel of the V21 monochromator and their product, determining the total transmitted intensity; without tuning, this total intensity is much lower than the peak intensities of the rocking curves.

Possible ways to compensate for the refractive angular shift of about 15′′ and thereby to increase the intensity throughput were summarized by Korytár *et al.* (2010 ▶); this paper also presented a preliminary study of a linearly graded GeSi V21 channel monochromator with both asymmetry angles of 15°. An intensity gain by a factor of more than six can be obtained compared with that of a reference pure Ge monochromator. By a simple calculation, the 15′′ fine tuning of the relative angular position of the two diffractors necessary to match the outgoing beam from diffractor 1 into the acceptance (incident) beam of diffractor 2 corresponds to

(*a*) 1.5 µm mechanical closing of the V-opening at a crystal length of 20 mm in mechanical tuning,

(*b*) a change of the Bragg angle due to a 0.3% higher Si content in Ge in position 2 compared with position 1,

(*c*) a temperature increase of Δ*T* = 30 K (for linear expansion coefficient α_Ge_ = 5.9 × 10^−6^ K^−1^) at position 1 relative to position 2.

We already proposed to compensate the refraction correction using a prism or multiprism, or a mirror in the first diffracted beam inside the channel.

The first results of a study of a thermally tuned reference V21 monochromator were presented by Áč *et al.* (2010 ▶): the intensity was more than five times higher compared with that of the case which had a zero temperature difference between the walls.

Another approach to match rocking curves inside a V-monochromator is based on unequal asymmetry angles of the two diffractors inside the channel. This type of V-monochromator is based on the fact that the refraction correction at an asymmetric diffractor at the non-grazing side is close to the refraction correction of the symmetrical or low asymmetry angle diffractor. It was designed and studied theoretically by Hart *et al.* (1995 ▶) and Servidori (2002 ▶). Ferrari *et al.* (2011 ▶) presented experimental results obtained in the study of a highly asymmetric V-channel monochromator with unequal asymmetry angles of 22.05 and 9.10° in beam expanding mode, with an eight-times higher flux compared with that of a symmetrical channel-cut monochromator (CCM). It should be noted that this type of a monochromator is very sensitive to (sub)surface defects and surface flatness when operated at high asymmetries.

A pair of crossed V-channel monochromators with unequal asymmetry angles of 19 and 4.28° (acting as a 15-fold de/magnifier V15 around 8 keV) were designed and studied for the purpose of a two-dimensional X-ray beam expansion or image magnification by Vagovič *et al.* (2011 ▶, 2012 ▶). With photon energy increased, this magnification factor can be increased over 150 times.

## 3. Thermal tuning of V21 monochromators

Temperature-dependent measurements of the X-ray beam intensity transmitted through the channel-cut crystals were carried out in a parallel beam geometry using a Bruker D8 DISCOVER diffractometer with a parabolic Göbel mirror in the primary beam.

**Figure 3:**
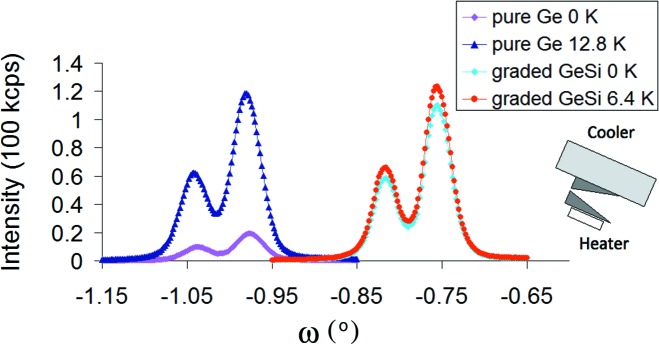
Experimental rocking curves from the reference pure Ge(220) and from the graded GeSi(220) V21 monochromators in beam compression mode. Heating/cooling sketched in the inset.

An electrical resistance heater and an aluminium cooler (inset in Fig. 3 ▶) were used to heat diffractor 1 of the V21 monochromator (Fig. 1 ▶) and to remove the heat supplied to diffractor 2 by thermal conduction, respectively. The temperature at the channel walls was measured remotely using an infrared sensor (Micro-Epsilon ThermoMETER CT-0F02-C3) with a sensitivity of 0.1 K.

The angular changes Δθ of the Bragg angle Θ_B_ were converted into the absolute value of the temperature difference Δ*T* and *vice versa* using a simple equation based on kinematical diffraction theory: 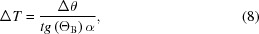
where α = 5.9E^−5^ K^−1^ is the linear thermal coefficient of expansion for Ge. It can be easily ascertained that Δ*T* (in K) is equal to 2Δθ in arcseconds in our case with Ge(220) diffractions and Cu *K*α radiation.

Fig. 3 ▶ shows experimental rocking curves from the reference pure Ge(220) and from the graded GeSi(220) V21 monochromators in beam compression mode.

**Figure 4:**
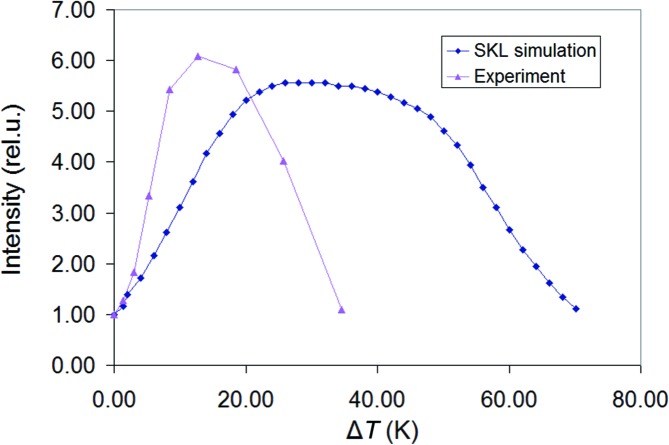
*SKL*-simulated temperature tuning curve (scan of transmitted beam peak intensity *versus* channel walls temperature difference) providing the region of the overlap of rocking curves inside the channel and experimental temperature tuning scan with a reference V21 monochromator in beam compressing mode.

A thermal tuning curve of a pure Ge V21 monochromator is presented in Fig. 4 ▶. A maximum increase of the transmitted peak intensity (about six times) was observed for a temperature difference of 12.8 K, which is its optimal tuning temperature. The FWHM of the thermal tuning curve was 24 K.

The graded GeSi monochromator was tuned through a composition gradient and the peak intensity was maximized by a minor translation of the crystal along the surface of diffractor 1 relative to the incident beam. The positional tuning was done before the thermal tuning. A 12% peak intensity increase was observed at a wall temperature difference of Δ*T* = 6.4 K. Fig. 4 ▶ also shows that the peak intensity of the transmitted beam through thermally and compositionally tuned V21 monochromators is comparable. Consequently, the effects of thermal and compositional lattice distortions are comparable. To make sure that the measured peak intensity was really the result of the optimal tuning, we replaced the cooler and the heater at diffractor 1 and 2 of the compositionally graded monochromator. As the temperature difference (in negative sense) increased, the peak of the transmitted intensity decreased. The FWHM of this thermal tuning curve was 26 K. The above measurement confirmed (within 12%) that the graded GeSi V21 monochromator was correctly designed.

Fig. 4 ▶ shows a comparison of the experimental and theoretical thermal tuning curve for a pure germanium V21 monochromator as simulated with the *SKL* beam tracing program (Mikulík & Kuběna, 2005 ▶). It demonstrates that the theoretical thermal tuning curve is slightly lower but two times broader than the experimental one (Table 1 ▶). Also, using a simple argument that if the FWHM = 27′′ and 6′′ of rocking curves from point 1 and 2 inside the channel are to be shifted and multiplied, the FWHM of the resulting curve approaching 30′ or 60 K is reasonable. Fig. 4 ▶ also shows that the experimental peak was scanned with Δ*T* faster than was expected and behaved so due to the lattice tilt or the accompanying curvature working in favour of the effect of the lattice parameter gradient (Smither *et al.*, 2005 ▶); this effect was not considered in the *SKL* simulation. The peak intensity increase is related to a zero temperature difference Δ*T* = 0 K. Its value of 1.12 for graded GeSi means the above is explained by the 12% mismatch. The experimental peak intensity gain is surprisingly even higher than the theoretical one and means more than six times intensity throughput at only 12.8 K temperature difference.

## 4. Comparison of the efficiency parameters of V-channel beam compressors

Standard symmetrical or asymmetrical channel-cut monochromators with parallel walls are beam conditioners that affect the monochromaticity and collimation of the beam. Therefore, they are also called monochrocollimators. A slit has to be used to condition the geometrical parameters, *e.g.* the beam width. The advantage of a V-channel beam compressor is its ability to provide monochromaticity, collimation and beam compression all in one device. There is another very important parameter related to the efficiency of the system – the throughput intensity, which is being compared together with the spectral resolution, beam divergence and beam width.

Table 2 ▶ shows a basic comparison of the microsource (IµS, Incoatec) with Montel optics, a V21 beam compressor and a slit (Vegso *et al.*, 2011 ▶): the main result is that the slit collimator gave a higher flux but did not remove the *K*α_2_ line.

Table 3 ▶ compares the intensity obtained by *SKL* simulations of the possible measuring setups. It shows that the slit-limited beam monochromated with a symmetrical Ge(220) CCM provided about 2.5× lower intensity than the V21 compressor. Using the asymmetric CCM with parallel walls allows for more intensity to come through the monochromator the larger the angle of asymmetry. The drawback is an increase in the divergence.

Table 4 ▶ shows the total flux and flux per 150 × 150 µm pixel measured with a two-dimensional silicon X-ray detector (Pilatus 100K, Dectris) working in the single-photon counting regime. It is demonstrated that the flux and also the flux per pixel were higher from the V21 monochromator than from a comparable slit with a symmetrical CCM. It is also shown that the imaging monochromator V15 (see *Theoretical background* [Sec sec2] and *X-ray beam expansion as two-dimensional image magnification* [Sec sec6.2]) used in the beam compressing mode gives, as a result of a larger size and acceptance, even higher intensity than V21.

## 5. Tuning by means of a plastic prism and multiprism

**Figure 5:**
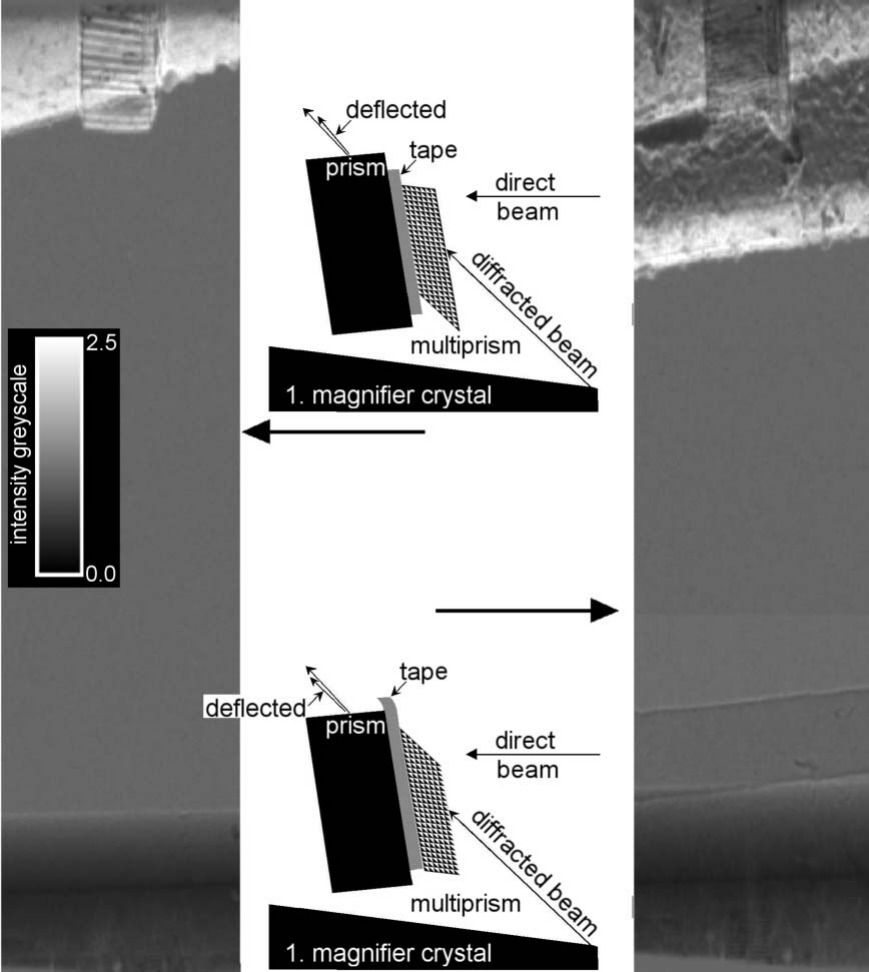
Vertically adjusted V21 monochromator as a beam expander with a single prism and multiprism inside the V-opening. The beam diffracted by the first diffractor is deflected upwards by the prism and multiprism (upper sketch). The lower sketch shows a flipped multiprism (rotated in its base by 180°) with the X-ray beam deflected again upwards by the prism, but downwards by the flipped multiprism. See text for further details.

The system operation can be simplified if the rocking curve matching is improved by deflecting the beam between the two diffraction processes independently. This can be achieved *via* tunable beam deflection using a tiny prism or multiprism, as was suggested by Korytar *et al.* (2010 ▶) for the tuning of V21 monochromators. In the present experimental conditions, the beam deflection could be as large as 2.5′′ in a single rectangular plastic prism, while it could be 17′′ in a multiprism for the correct angular adjustment (Jark *et al.*, 2004 ▶). The related experimental verification was made on the Optics beamline at the ESRF, Grenoble, using a beam monochromated by a double-crystal Si(111) monochromator to 8 keV. A multiprism was attached to a simple plastic prism (Fig. 5 ▶), which was mounted on a second goniometer, such that the combination could be introduced independently in the beam path between the two beam diffractions in the V21 monochromator. Consequently, the deflection angle of the once-diffracted beam could be varied. The X-ray images of the prism and multiprism, which were only magnified by the second diffractor of the V-monochromator, were taken using a FReLoN camera depending on their angular position in a scan covering a total of 22°. Fig. 5 ▶ shows the experiment setup for both orientations of the multiprism, when the beam diffracted by the first diffractor is additionally deflected by the prism and the multiprism. The corresponding X-ray images are shown to the left (upper configuration) and to the right (lower configuration). The single prism has the same orientation in both configurations and consequently it provided the projected beam deflection in both cases. The deflection angle varied little in the angular scan and consequently this prism already provided an intensity increase (white) of about two- to threefold, though a larger increase should have been possible with larger beam deflection. The latter should have been achieved in the multiprism, when mounted in the configuration from the upper plot. It is now understood that the scan did not cover the correct orientation angle and consequently the performance did not meet with expectations.

Nevertheless, even the misaligned multiprism provided an intensity gain of about two- to threefold. Instead, when mounted for increasing the mismatch, the multiprism should provide an intensity decrease. This expected behaviour will make the multiprism appear as a darker shadow in the beam, which is enhanced in the simple prism. This agrees with the observation in the right image.

In summary, we investigated the concept of tuning a V-monochromator by means of prism structures. Some gain was observed, when operating a single prism with a smaller deflection angle. In this case the deflection was insufficient, while the increasing absorption in the thicker part of the prism restricted the use of single prisms providing larger deflection to rather small beams. Multiprisms are more efficient for both aspects, as these more transparent structures should also provide larger deflection angles. The beam deflection in these structures was in the expected direction, though it was smaller than expected owing to inappropriate alignment. The observed intensity enchancement agrees with the expectations for a misaligned multiprism, and consequently the present structure should be capable of providing better matching between the two diffraction processes in future experiments with better alignment.

## 6. Metrological and imaging applications

### 6.1. Application in GISAXS

**Figure 6:**
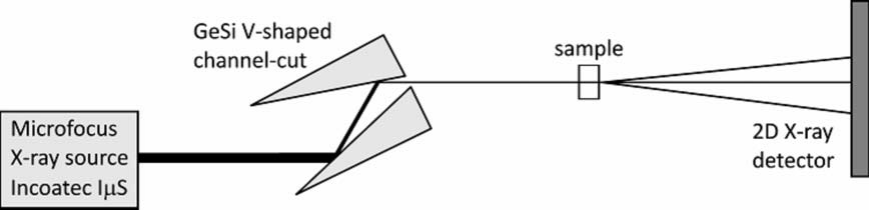
Top view of the experimental setup comprising a microfocus X-ray source with integrated Montel optics and a V-shaped beam compressor.

**Figure 7:**
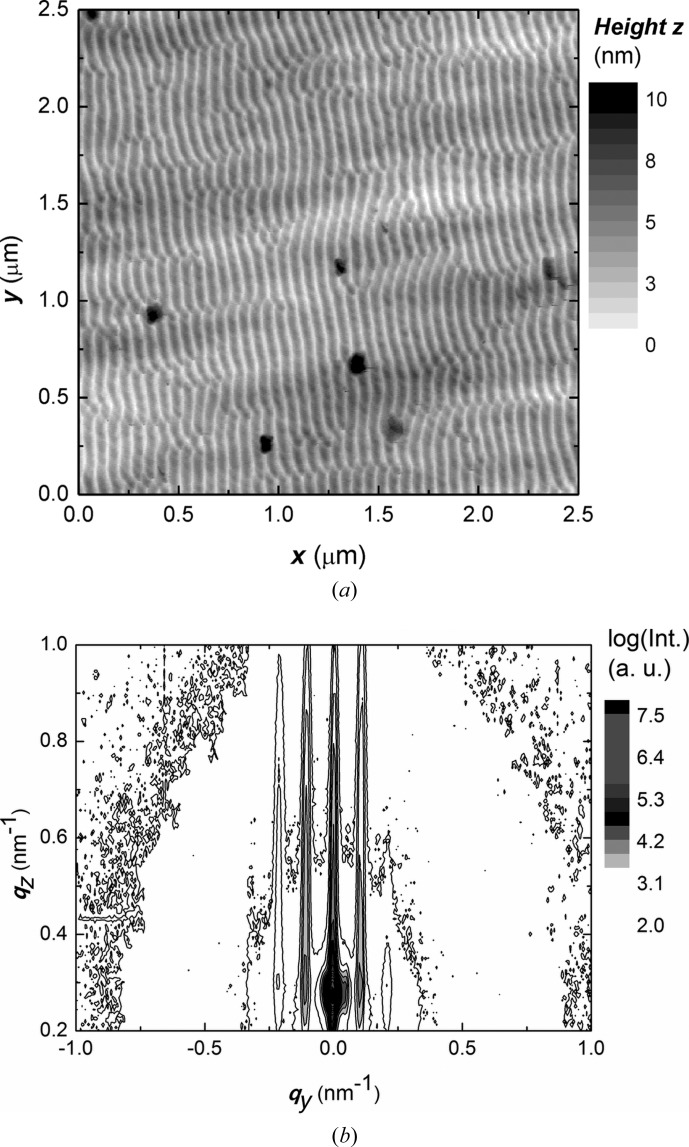
(*a*) Ripple formations obtained at the Si surface corrugated with ion beam sputtering and observed by AFM (Dimension Edge, Bruker AXS) in the tapping mode (cantilever OTESPA, Bruker AXS). (*b*) Corresponding GISAXS map with truncation rods.

A graded V21 compressor was integrated into a home-made GISAXS setup (Fig. 6 ▶) consisting of a microfocus Cu *K*α X-ray source (IµS, Incoatec) with collimating Montel optics producing a nearly parallel beam with divergence 500 µrad, dimensions 1.3 × 1.3 mm and flux 3 × 10^8^ counts s^−1^. The V21 monochromator was placed at a distance of 58 cm from the X-ray source in the centre of a high-precision goniometer with bi-directional repeatibility 0.001°. The lateral size of the X-ray beam on the monochromator of 1.3 × 0.058 mm suggests a compression factor of 22 which is a value slightly larger than the theoretical one. The flux behind the compressor, measured with a two-dimensional silicon X-ray detector (Pilatus 100K, Dectris) working in the single-photon counting regime, was 1.1 × 10^6^ counts s^−1^. An Si sample with its surface corrugated by ion-beam sputtering was adjusted to have an angle of incidence of 0.2°. The scattered X-ray photons were also collected by a Pilatus detector. The GISAXS pattern was integrated for 1 h. The sample–detector distance was set to 1 m. The GISAXS pattern obtained is shown in Fig. 7 ▶ *b*). It reveals several truncation rods running along the *q*_*z*_ axis. The mean positions of the adjacent lateral maxima were evaluated and the mean lateral spacing was calculated. The lateral periodicity was determined as *d* = 2π/Δ*q*_*y*_ = 58.600 (12) nm. For comparison, the surface morphology was also re-measured by a commercial atomic force microscope (Dimension Edge, Bruker AXS) in tapping mode (cantilever OTESPA, Bruker AXS). The power spectral density of the surface morphology of the silicon grating in the lateral direction was then evaluated from the atomic force microscopy (AFM) image. The AFM measurement (Fig. 7 ▶ *a*) provided a value of 61.6 (6) nm. Details concerning the sample preparation method and periodicity analysis were given by Siffalovic *et al.* (2010 ▶). The difference between the GISAXS and AFM measurements can be explained by the significantly local character of the AFM measurement while GISAXS provides averaging across the sample surface (Renaud *et al.*, 2009 ▶). In order to determine the instrumental resolution of the GISAXS setup, the zeroth truncation rod at *q*_*y*_ = 0 nm^−1^ was fitted with a Gaussian function in the lateral direction. The FWHM of the central peak was 0.0117 (4) nm^−1^. As a consequence, the instrumental resolution of the GISAXS system was determined to be Γ = 2π/FWHM = 534.3 (27) nm. The estimated value of the lateral resolution points to an excellent performance by the developed device, which thus offers a useful alternative to the traditional double-pinhole system (Vegso *et al.*, 2011 ▶).

### 6.2. X-ray beam expansion as two-dimensional image magnification

Fig. 5 ▶ shows an X-ray image of a prism and multiprism magnified by the second asymmetric diffractor of the V21 monochromator (magnification *m*_2_ = 4.6) in the vertical dimension (one-dimensional magnification). Putting an object into the incident beam it is possible to obtain a one-dimensional 21-fold beam expansion or image magnification.

**Figure 8:**
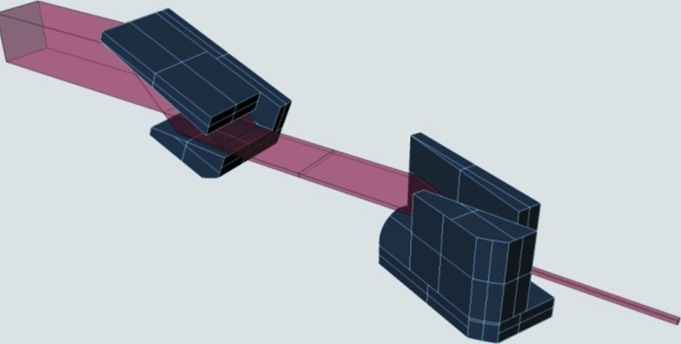
Principle of the two-dimensional in-line Bragg de/magnifier based on crossed V-channel monochromators. For beams incident from the left side it acts as a two-dimensional beam compressor and for those from the right side as a two-dimensional expander. The magnification is 15 for photon energy around 8 keV.

**Figure 9:**
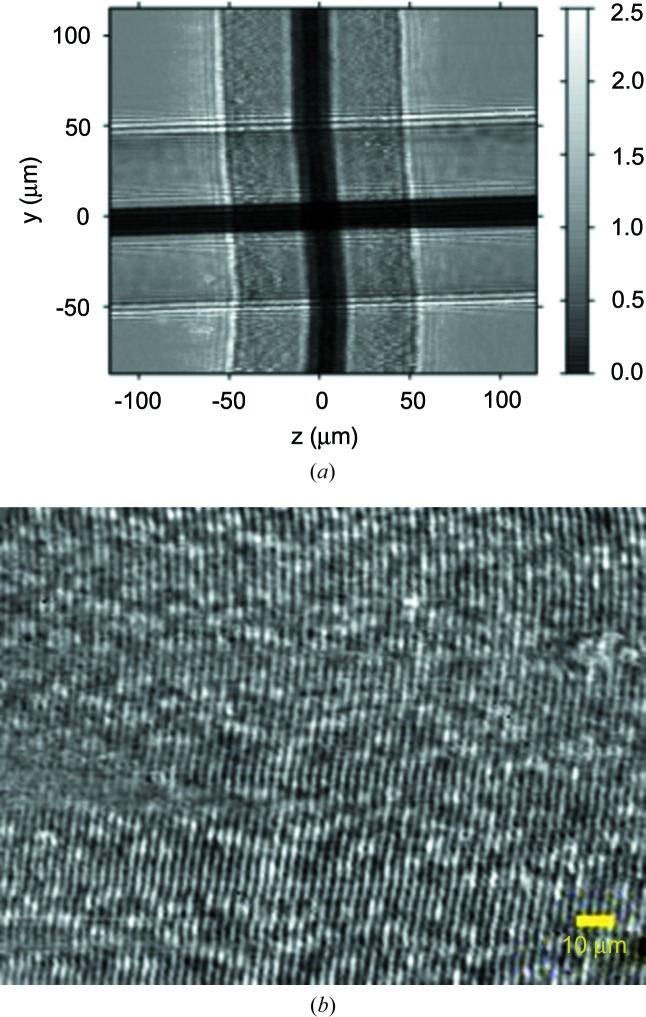
The image of a crossed boron–tungsten fibre with the outer diameter of 100 µm and with the tungsten core diameter of 14 µm (*a*) and the image of a 2.4 µm-period grating of SU-8 resist on Kapton foil. The images were obtained with the two-dimensional Bragg magnifier from Fig. 8 ▶ at an energy of 9.15 keV (the effective pixel size is below 1 µm).

Fig. 8 ▶ depicts the principle of the two-dimensional in-line Bragg de/magnifier based on crossed V-channel monochromators. In beam expanding mode, it can effectively increase the spatial resolution of the camera by a factor of magnification and thus decrease the effective pixel size by about the same value. The combination of this Bragg magnifier with a FReLoN camera and a two-dimensional Medipix silicon-based camera working in single-photon counting mode was studied by Vagovič *et al.* (2011 ▶, 2012 ▶, 2013 ▶) with several test structures (Siemens star, Cu grids of different openings and biological objects) with the aim of exploring the imaging possibilities in absorption and phase-contrast modes. Fig. 9 ▶(*a*) shows the image of another test structure – a crossed boron–tungsten fibre with an outer diameter of 100 µm and with a tungsten core diameter of 14 µm. The image was obtained at the SLS Optics beamline X05DA at a photon energy of *E* = 9.15 keV with a Medipix camera. For this energy the horizontal (vertical) magnification reached the value of 59 (50) for which the effective horizontal (vertical) pixel size is 0.93 µm (1.09 µm). In the experimental conditions applied, the interference at the boron edge is observable even at a propagation distance as short as 140 mm. Fig. 9 ▶(*b*) shows the image of a 2.4 µm-period grating of SU-8 resist on Kapton foil and demonstrates that the effective pixel size of the Bragg magnifier–Medipix camera system is as low as about 1 µm.

The crossed V15 Bragg magnifier (magnification *M* = 15 for Cu *K*α_1_ radiation) combined with a direct converting Medipix camera was shown to also work reliably in laboratory conditions, using a microfocus source with a Cu anode and with collimating Montel optics, reaching a spatial resolution below 10 µm (Vagovič *et al.*, in preparation).

In conclusion, several ways of tuning V-channel monochromators with higher asymmetries were studied, and some results obtained in one metrological and two imaging applications were presented.

## Figures and Tables

**Table 1:** Parameters of theoretical and experimental thermal tuning curves of the germanium-based V21 monochromators

	Δ*T*(K)	Intensity gain(relative units)	FWHM(K)
*SKL* simulations	26	5.56	50
Pure Ge V21 (experiment)	12.8	6.08	24
Graded GeSi V21 (experiment)	6.4	1.12	26

**Table 2:** Comparison of parameters from a one-dimensional asymmetric compressor and slit collimator with Montel optics

Parameter	Montel optics	V21 compressor	Slit collimator
Flux (counts s^−1^)	10^8^	1.1 × 10^6^	1.5 × 10^6^
Dimensions (mm)	2 × 2	2 × 0.1	2 × 0.1
Divergence (mrad)	0.5 × 0.5	0.5 × 0.07	0.5 × 0.1
Spectral resolution (δλ/λ)	5.3 × 10^−2^	1.6 × 10^−3^	5.3 × 10^−2^

**Table 3:** Simulated experimental setup for a microsource and Montel optics (rectangular source with divergencies 0.028 × 0.05°)

Simulated setup	Integrated intensity (counts s^−1^)
Slit 0.1 mm	77900
Graded GeSi V21 CCM	11030
Slit + sym 2× Ge(220) CCM	4390
Slit + asym (+15°, −15°) Ge(220) CCM	8790
Slit + asym (+17°, −17°) Ge(220) CCM	10070

**Table 4:** Comparison of the intensity throughput (Pilatus 100K, Dectris) in several experimental setups

Experimental setup	Flux(counts s^−1^)	Flux per pixel150 µm^2^(relative units)	Notes
Montel optics	1 × 10^8^	N.A.	*K*α_1,2_ present
Montel + slit 40 µm	4.7 × 10^6^	N.A.	*K*α_1,2_ present
Montel + slit 40 µm + symGe(220) CC	1.4 × 10^5^	2.5 × 10^4^	*K*α_1_
Montel + V21 CC, beam width 58 µm	3.2 × 10^5^	6.3 × 10^4^	*K*α_1_, 4× highercollimation
Montel + V15 CC	1.08 × 10^6^	1.68 × 10^5^	Higher acceptance, 2× longer
